# Systematic Review of Paraspinal Muscle Changes in Lumbar Spondylolisthesis: MRI and CT Insights

**DOI:** 10.1111/os.70362

**Published:** 2026-07-14

**Authors:** Valerio D'Andrea, Caterina Bernetti, Federico Greco, Claudia Volterrani, Carlo C. Quattrocchi, Paul M. Parizel, Johan Van Goethem, Gianfranco Di Gennaro, Bruno Beomonte Zobel, Carlo A. Mallio

**Affiliations:** ^1^ Fondazione Policlinico Universitario Campus Bio‐Medico Rome Italy; ^2^ Research Unit of Radiology, Department of Medicine and Surgery Università Campus Bio‐Medico di Roma Rome Italy; ^3^ Casa di Cura Villa Stuart Rome Italy; ^4^ Department of Radiology Cittadella Della Salute Azienda Sanitaria Locale di Lecce Lecce Italy; ^5^ Centre for Medical Sciences‐CISMed, University of Trento Trento Italy; ^6^ Royal Perth Hospital (RPH) Perth Australia; ^7^ Medical School, University of Western Australia (UWA) Perth Australia; ^8^ Department of Radiology Antwerp University Hospital Edegem Belgium; ^9^ Department of Health Sciences Chair of Medical Statistics, University of Catanzaro “Magna Græcia” Catanzaro Italy

**Keywords:** cross‐sectional area, CT, MRI, paraspinal muscles, spondylolisthesis

## Abstract

**Background:**

Spondylolisthesis is a frequently encountered condition in orthopedics, requiring dynamic stability maintained primarily by the paraspinal muscles. This systematic review evaluates the cross‐sectional area (CSA) and fat infiltration (FI) of paraspinal muscles—specifically the multifidus, erector spinae, and psoas major—in lumbar spondylolisthesis (LS) to understand their roles in spinal stability and progression in degenerative and isthmic forms.

**Methods:**

Following PRISMA guidelines, PubMed, Cochrane, and Scopus were searched as of September 2024. Fourteen retrospective studies using MRI/CT imaging met inclusion criteria, focusing on CSA and FI in LS. Data were extracted from studies that assessed spinal instability through vertebral slippage measurements or comparisons with asymptomatic controls.

**Results:**

The findings indicate that reduced CSA and increased FI in paraspinal muscles, especially the multifidus, are associated with spinal instability and progressive vertebral slippage. Several studies reported reductions in CSA and increases in FI in patients with spondylolisthesis, although methods and anatomical levels varied across studies. Compensatory hypertrophy of the erector spinae and psoas major was observed, particularly in isthmic cases.

**Conclusions:**

These findings support the potential role of muscle health in maintaining spinal stability and suggest that targeted rehabilitation strategies addressing paraspinal muscle alterations may improve clinical outcomes in patients with LS.

## Graphical abstract

Paraspinal muscle degeneration, especially multifidus atrophy and fat infiltration (FI), contributes to spinal instability and spondylolisthesis progression. Reduced multifidus area and increased FI correlate with instability, while compensatory erector spinae hypertrophy is often observed, particularly in isthmic cases, highlighting the need for targeted rehabilitation.

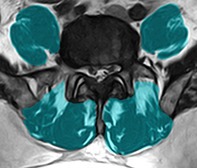

**Abbreviations**
AUC: area under the curve
CSA: cross‐sectional area
DHI: disc height index
dl‐DLS: double‐level DLS
DLK: degenerative lumbar kyphosis
DLS: degenerative lumbar spondylolisthesis
ES: erector spinae
ES‐LCSA: erector spinae lean cross‐sectional area
FI: fat infiltration
FIR: fat infiltration ratio
iDHI: initial disc height index
IS: isthmic spondylolisthesis
LL: lumbar lordosis
LSS: lumbar spinal stenosis
MF: multifidus
M‐LCSA: multifidus lean cross‐sectional area
OR: odds ratio
PI: pelvic incidence
PM: psoas major
PT: pelvic tilt
sl‐DLS: single‐level DLS
SMM: skeletal muscle mass
SS: sacral slope

## 1 Introduction

Spondylolisthesis, derived from the Greek “spondylos” (vertebra) and “olisthesis” (to slip or slide down an incline), refers to the forward or backward displacement of a vertebra and has increasingly become a frequently encountered condition in orthopedics [1, 2]. Its prevalence varies by region based on racial and genetic factors, with earlier studies reporting rates of 22.5% in China and 21.2% in the United States [3, 4]. Lumbar spondylolisthesis (LS) may be triggered by dysplasia, isthmic fissures, degeneration, trauma, and pathological conditions, with isthmic (IS) and degenerative (DLS) types being the most prevalent forms [3]. The paraspinal muscles are crucial for maintaining an upright posture and ensuring the spine's dynamic stability, playing a key role in both the development and compensation of LS [5]. Medical imaging offers non‐invasive and reproducible data [6, 7] on muscle density, cross‐sectional area (CSA), and other muscle properties, including fat infiltration (FI) [3, 8]. Lumbar muscles, including the psoas major (PM), erector spinae (ES), and multifidus (MF) muscles, are vital for the stability and functional movement of the lumbar spine. In the present review, the psoas major was included due to its recognized role in lumbar stabilization and spinopelvic biomechanics. Moreover, the ES muscle group, which includes the iliocostalis, longissimus, and spinalis muscles, contributes to spinal extension and the maintenance of an upright posture. To assess the size of these muscles, CSA can be reliably measured using either computed tomography (CT) or magnetic resonance imaging (MRI) [6, 9, 10]. Paraspinal muscles imaging characteristics, such as CSA and density (CT) or signal intensity (MRI), are impacted by multiple factors, such as aging, physical fitness, diet, body weight, and the presence of back pain [11, 12, 13, 14]. Strength and condition of paraspinal muscles are key to sustaining proper spinal alignment and balance [15, 16].

Assessing CSA of paraspinal muscles in LS patients can reveal the biological processes and compensatory mechanisms involved, aiding in developing individualized treatment and rehabilitation plans to improve outcomes and quality of life [17].

This systematic review aims to evaluate CSA and FI of lumbar muscles (MF, ES, and PM) in patients with LS, focusing on imaging studies, to clarify the relationship between muscle morphology, spinal stability, and the progression of DLS and IS.

## 2 Materials and Methods

### 2.1 Search Strategy

This systematic review followed the PRISMA (Preferred Reporting Items for Systematic Reviews and Meta‐Analyses) guidelines. The literature search was performed on September 30, 2024, across the following electronic databases: PubMed, Cochrane Library, and Scopus. The search utilized a combination of the keywords “spondylolisthesis,” “paraspinal muscles,” “multifidus,” “erector spinae,” “psoas,” “magnetic resonance imaging (MRI),” and “computed tomography (CT)” using the following search strategy: (“spondylolisthesis”) AND (“paraspinal muscles” OR “psoas” OR “multifidus” OR “erector spinae”) AND (“cross‐sectional area”).

Google Scholar and reference lists were also searched. No date restrictions were applied.

The protocol for this systematic review has been registered on The Open Science Framework Registrie (OSF) (DOI: 10.17605/OSF.IO/SGY3W) [18].

### 2.2 Study Selection

After duplicates removal, titles and abstracts were screened. Full texts were assessed using these criteria: (1) spondylolisthesis and paraspinal CSA evaluation; (2) MRI or CT imaging; (3) participants ≥ 18 years; (4) full text in English. Excluded were reviews, preclinical studies, case reports, non‐lumbar evaluations, and post‐operative follow‐up studies assessing muscle morphology after surgical intervention.

**FIGURE 1:**
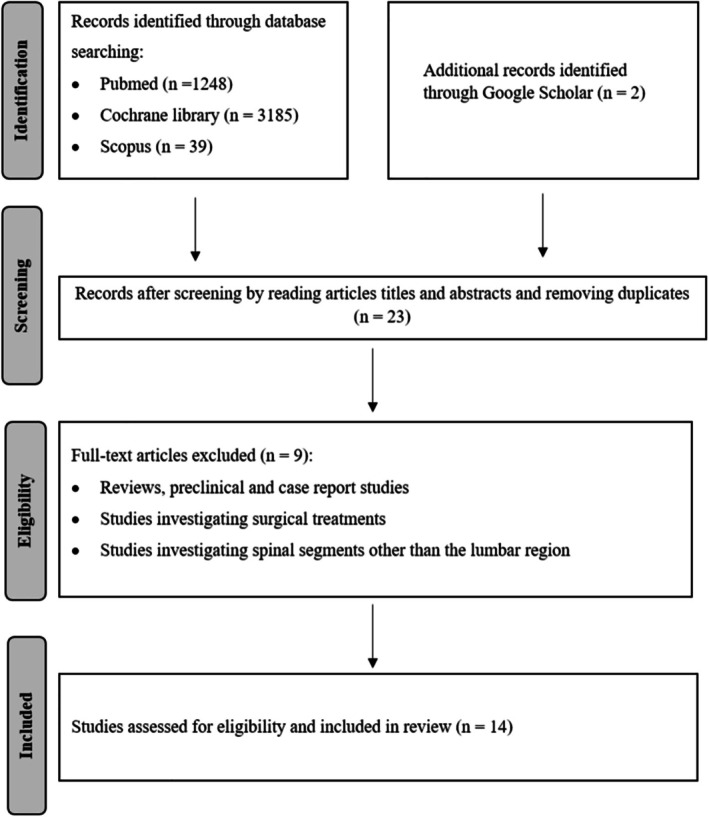
PRISMA diagram showing the flow of studies selection through phases of the review.

Selection is shown in Figure 1.

### 2.3 Data Extraction

**FIGURE 2:**
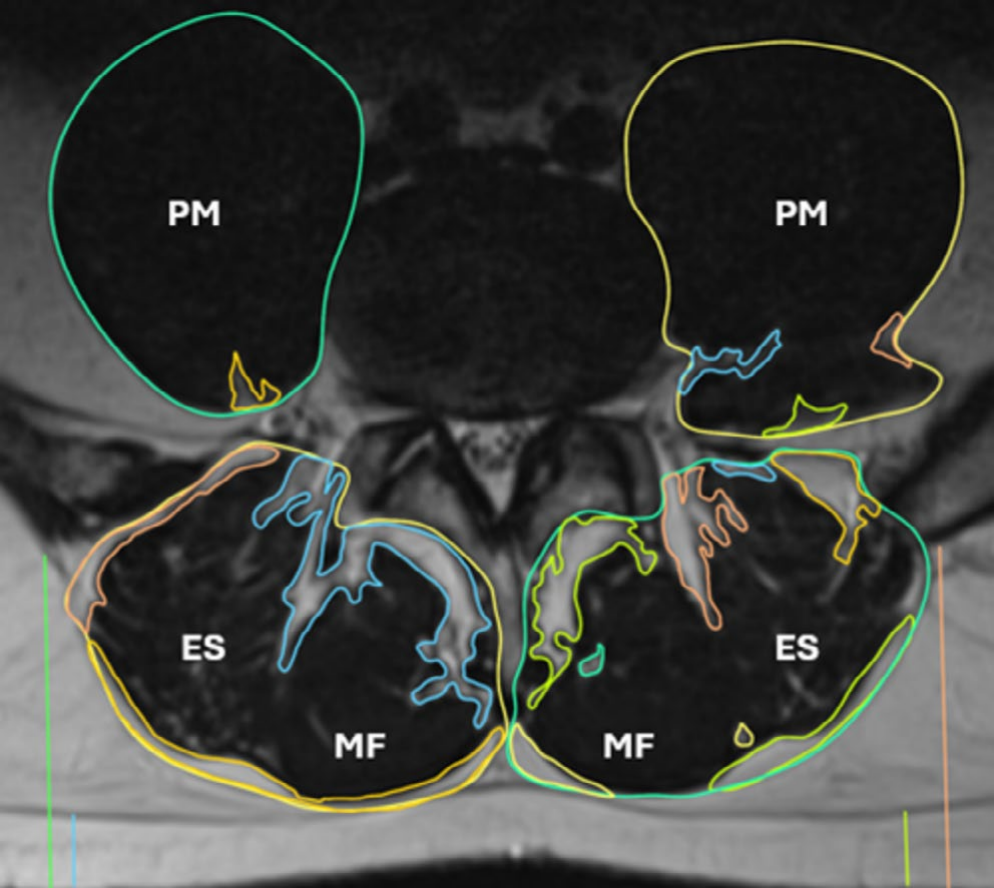
Axial T2‐weighted MRI image at the L4–L5 intervertebral disc level. The image shows the segmentation of the paraspinal and PM, highlighting their CSA. Intramuscular FI is visually represented within muscle contours. A perpendicular line was drawn from the lateral margin of the paraspinal muscle to the dermal boundary to illustrate the measurement of posterior subcutaneous fat thickness. MF: Multifidus; ES: Erector spinae; PM: Psoas major.

Study selection, full‐text screening, and data extraction were performed independently by two reviewers. Disagreements were resolved through discussion and consensus with a third senior reviewer. The extracted data were: authors, year, study design, demographics, imaging modality, parameters, key findings. For each study, design, imaging (MRI/CT), muscles (MF, ES, PM), and CSA/FI measurement (manual or threshold‐based) were recorded. Data were tabulated and grouped by muscle changes (atrophy, hypertrophy, or FI) and their link to LS. A visual example is in Figure 2. Due to substantial methodological and quantitative heterogeneity across the included studies, spanning imaging modality (MRI vs. CT) and primary parameter derivation (e.g., quantitative FI vs. semiquantitative grading), a structured narrative synthesis (SWiM) was conducted using the following explicit rules:

- Grouping and Direction‐of‐Effect: Findings were systematically grouped by muscle type (MF, ES, PM) and morphological parameters (CSA vs. FI) to assess the consistent direction of effect (e.g., reduction in MF CSA, increase in ES hypertrophy/CSA).
- Handling Heterogeneity: The synthesis prioritizes findings showing statistical significance (*p* < 0.05) within a consistent direction of effect. Non‐significant findings were reported transparently and discussed in the context of the study's specific methodology. Detailed methodological differences (e.g., normalization methods, specific lumbar levels measured) are systematically summarized in Table 2.

The methodological quality of the 14 included studies was assessed using the Newcastle–Ottawa Scale (NOS), which is suitable for evaluating the quality of non‐randomized observational cohort studies (Table S1).

## 3 Results

### 3.1 Study Selection and Characteristics

The systematic search yielded 4472 articles, plus 2 from manual search. After removing duplicates and screening titles, abstracts, and full texts, 14 studies met the inclusion criteria. All studies were retrospective and used MRI or CT imaging; some also included radiographic projections. Study characteristics are reported in Table 1.

**TABLE 1:** Summary of the 14 studies included in the systematic review. All the studies were retrospective.

Author, (Year)	Sample size (Case/Control)	Control group description	Diagnosis and Grading	Main outcomes related to CSA (abbreviated)
Wang et al. (2015) [19]	149 total	Healthy controls (age/sex matched)	L4/L5 DLS. Graded.	MF LCSA ↓ in DLS. ES LCSA ↑ in DLS.
Wagner et al. (2018) [20]	101 total	Internal: Mild/Moderate Disability Group	DLS (Grade I or II).	PM CSA ↓ in severe disability (*p* = 0.041).
Hiyama et al. (2019) [21]	288: 140 total (full analysis)	N/A (Internal correlation analysis)	LSS and/or DLS. Graded.	PM/MF CSA correlated strongly with SMM and PT.
Park et al. (2019) [22]	219: DLS: 125/IS: 94	N/A (Internal DLS vs. IS comparison)	DLS vs. IS (Diagnosis by Radiologist).	MF/PM CSA negatively correlated with SP in IS only (*p* < 0.05).
Lee et al. (2021) [23]	62: DLS: 30/Non‐DLS: 32	Non‐DLS patients with chronic radiculopathy (Age/Sex matched)	DLS (L4/L5, 96.6% Grade I).	MF FCSA ↓/FI ↑ in DLS. ES FCSA ↑ in DLS.
Ohyama et al. (2021) [24]	50: DLS: 25/Non‐DLS: 25	Non‐DLS patients (Age/Sex matched via PSM)	L4 DLS (all Grade I).	Lower FI in PM/ES in DLS vs. non‐DLS (*p* < 0.05).
Li et al. (2022) [25]	243: IS: 81/DLS: 78/Control: 84	Healthy adults (Age matched)	IS vs. DLS vs. Healthy (L4 single‐segment, Grade I).	TCSA/VCSA ↓ (Control > IS > DLS, *p* < 0.05). FI higher in DLS.
Wang et al. (2022) [26]	149 total	Healthy controls (Age/Sex matched).	DLS (L4/L5) vs. Healthy.	RCSA of MF, ES, and PM ↓ in DLS (*p* < 0.05).
Ding et al. (2022) [27]	154 total	N/A (DLK internal comparison)	DLS vs. DLK. Graded.	MF atrophy more pronounced in DLS (*p* < 0.05).
Yang et al. (2023) [28]	160 total	N/A (Internal correlation analysis)	DLS and LSS. Graded.	Higher FI correlated with greater PT and PI (*p* < 0.001).
Cao et al. (2023) [29]	128: DLS: 60/Control: 68	Asymptomatic adults without DLS.	L4 DLS (90% Grade I).	PM FIR independent risk factor for L4 DLS (OR = 3.746, *p* = 0.038).
Li et al. (2024a) [17]	111: DLS: 37/IS: 37/Control: 37	Healthy adults (Age/Sex/BMI matched via PSM)	DLS vs. IS vs. Healthy (slip < 50%).	MF rfCSA ↓ in DLS/IS. ES rfCSA ↑ in IS only (*p* < 0.05).
Liu et al. (2024) [30]	220: DLS: 110/Control: 110	Healthy controls (Age/Sex matched)	Single‐segment DLS.	MF/ES/PM r‐CSA ↓/FIR ↑ in DLS (*p* < 0.001). MF/PM FIR independent risk factors.
Li et al. (2024b) [31]	140: dl‐DLS: 70/sl‐DLS: 70	N/A (Internal dl‐DLS vs. sl‐DLS comparison)	Double‐level DLS (dl‐DLS) vs. Single‐level DLS (sl‐DLS).	MF/ES/PM rCSA ↓/FI ↑ more severe in dl‐DLS (*p* < 0.001).

Abbreviations: CSA, cross‐sectional area; ES, erector spinae; FCSA, functional cross‐sectional area; FI, fat infiltration; LCSA, lean cross‐sectional area; MF, multifidus; PM, psoas major; rCSA, relative cross‐sectional area; SP, slip percentage; TCSA, total cross‐sectional area.

**FIGURE 3:**
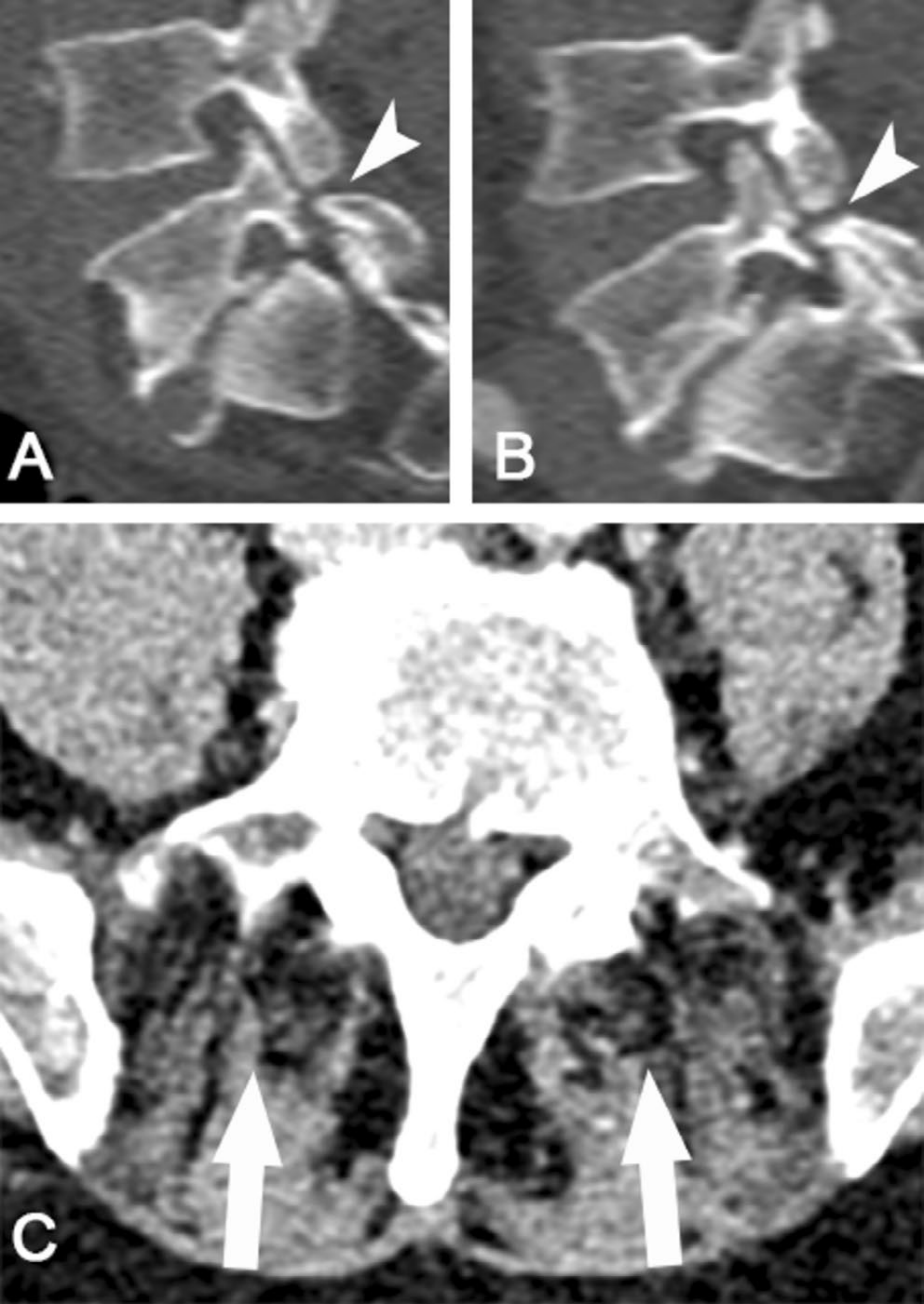
Sagittal (A‐B) and axial CT images (C) of L5‐S1 IS. The bilateral isthmic lysis presents anterolisthesis of the L5 vertebral body over S1 (arrowheads in A‐B). The axial image (C) shows diffuse moderate atrophy and FI of the paravertebral muscles, particularly of the MF (arrows in C).

Findings suggest reduced CSA and increased FI, especially in the MF, may contribute to spinal instability and slippage progression (Figure 3).

Several studies [17, 19, 25, 27, 30] reported reductions in CSA and increases in FI in LS patients, although methods and anatomical levels varied across studies. Compensatory hypertrophy in the ES and PM is notably observed in cases of IS [32, 33]. Methodological details are summarized in Table 2.

**TABLE 2:** Summary of the methodology details.

Author, (Year)	Imaging modality (Sequence)	Lumbar levels measured	FI measurement method/Thresholds	CSA normalization method	Other measures/Reliability
Wang et al. (2015) [19]	MRI (T2‐weighted axial)	L3–L5	Signal Intensity Ratios (RM and RES)	VB CSA	High ICCs (0.83–0.95)
Wagner et al. (2018) [20]	MRI (T1‐weighted axial)	L3–L4 disc midpoint	N/A (Only CSA assessed)	Total Paraspinal CSA	Two blinded reviewers.
Hiyama et al. (2019) [21]	MRI (T2‐weighted axial)	L3–L4, L4–L5, L5–S1 disc levels	N/A (Only CSA assessed)	N/A (Absolute CSA/AveCSA used)	Excellent ICCs (> 0.90)
Park et al. (2019) [22]	MRI (T2‐weighted axial)	L5 upper endplate	N/A (Only CSA assessed)	VB CSA (L5)	Excellent ICCs reported
Lee et al. (2021) [23]	MRI (T2‐weighted axial)	L4 inferior endplate	Thresholding; FI = 100%‐FCSA/Total CSA%	VB CSA (rCSA); Patient height (SMI)	High ICCs reported
Ohyama et al. (2021) [24]	CT	L4–L5 disc level	FCSA/GCSA ratio (FI = GCSA‐FCSA/GCSA)	VB CSA (L4 pedicle level)	High precision and reproducibility reported
Li et al. (2022) [25]	MRI (T2‐weighted axial)	L4–L5 disc mid‐plane	Visual grading (Grade 0: < 10%; Grade 3: > 50%)	VB CSA	High reproducibility reported
Wang et al. (2022) [26]	MRI (T2‐weighted axial) and CT	L4–L5 disc mid‐plane	Grayscale intensity on T2‐weighted images (ImageJ)	VB CSA	High agreement reported
Ding et al. (2022) [27]	MRI (T2‐weighted axial)	L1–S1 (parallel to sup. endplate)	ImageJ Threshold (Grayscale value of 120 for fat)	VB CSA	High ICCs (> 0.96)
Yang et al. (2023) [28]	MRI (T2‐weighted axial)	L1–S1 (mid‐plane of disc)	ImageJ (8‐bit binary conversion for segmentation)	Total Muscle CSA (FI ratio)	High ICCs (> 0.90)
Cao et al. (2023) [29]	CT	L4–L5 disc level	HU Thresholds (Low‐Atten. Muscle: −29 to 29 HU; Adipose: −190 to −30 HU)	FI calculated as ratio (FIR, LTR)	High reproducibility reported
Li et al. (2024a) [17]	MRI (T2‐weighted axial)	L3–S1 disc mid‐plane	Thresholding based on grayscale values (FI ratio)	VB CSA (rCSA, rfCSA)	High ICCs (> 0.90)
Liu et al. (2024) [30]	MRI (T2‐weighted axial)	L3/4, L4/5, and L5/S1 disc levels	Threshold techniques on grayscale T2‐weighted images	VB CSA (r‐CSA)	High ICCs (> 0.90)
Li et al. (2024b) [31]	MRI (T2‐weighted axial)	L1–S1 disc mid‐plane	Threshold method for grayscale differentiation (FI ratio)	VB CSA (rCSA)	Excellent ICCs (> 0.90)

Abbreviations: CSA, cross‐sectional area; CT, computed tomography; DICOM, digital imaging and communications in medicine; ES, erector spinae; FCSA, functional cross‐sectional area; FI, fat infiltration; GCSA, gross cross‐sectional area; HU, Hounsfield unit; IC, iliocostalis; ICC, intraclass correlation coefficient; LCSA, lean cross‐sectional area; LG, longissimus; MF, multifidus; MRI, magnetic resonance imaging; PM, psoas major; PS, psoas; rCSA, relative cross‐sectional area; RES, relative signal intensity; RM, reference muscle; ROI, region of interest; SMI, skeletal muscle index; SP, slip percentage; SPSS, statistical package for the social sciences; TCSA, total cross‐sectional area; VB CSA, vertebral body cross‐sectional area; VB, vertebral body.

Only a subset of studies distinguished Functional CSA (FCSA), lean muscle excluding fat, from Gross CSA (GCSA) [17, 23, 24, 27, 28], providing a more detailed perspective on muscle quality by assessing the proportion of lean muscle relative to total muscle. Most studies utilized MRI as the primary imaging modality; whereas two studies [24, 29] employed CT to quantify fat content using Hounsfield Units (HU) [17, 18, 19, 23, 25, 27, 28, 30, 32, 33]. MRI‐based studies [17, 19, 20, 21, 22, 23, 26, 27, 28, 30] used grayscale thresholding or signal intensity ratios for FI analysis, mainly exploiting T2‐weighted axial sequences, which provide excellent soft tissue contrast for differentiating muscle and fat, except for one study that used T1‐weighted axial sequences for CSA evaluation [29].

Additionally, one study [25] implemented a visual grading system for FI, categorizing it into four grades (0: < 10%, 1: 10%–25%, 2: 25%–50%, 3: > 50%), offering a semi‐quantitative approach.

Some studies restricted measurements to a single lumbar level [25, 26, 27, 28, 29], while others analyzed multiple segments, covering the full L1 to S1 range or specific parts [17, 19, 21, 22, 23, 24, 27, 30].

The muscles studied consistently included the MF, ES, and PM, reflecting their critical role in lumbar spine stability and degenerative conditions. Reliability was key in all studies, with Intraclass Correlation Coefficients (ICCs) validating measurement consistency. Several studies [17, 19, 21, 23, 24, 27, 30] explicitly reported ICC values above 0.90, confirming high reproducibility, while others [20, 22, 25, 26, 29] described ICCs as high or excellent without exact values, indicating strong interobserver and intraobserver agreement.

Most studies normalized CSA values to vertebral body CSA (VB CSA) to adjust for body size differences [17, 20, 21], or patient height, calculating the Skeletal Muscle Index (SMI) for size‐adjusted metrics [18, 22, 26]. Software tools varied across studies: imageJ was widely used [18, 19, 21, 22, 23, 27, 30] for ROI tracing and FI analysis, while PACS systems [28, 29, 31] facilitated image storage and preliminary analysis. Meanwhile, workstation‐specific tools were employed in CT‐based studies, including ZIO station [26] and GE AW 4.6 [28].

## 4 Discussion

### 4.1 Paraspinal Muscle Morphology and Clinical Implications

To enhance the transparency and clinical interpretability of our results, we have evaluated the certainty of evidence for the key associations using the GRADE system. Given the prevalence of retrospective and observational studies, the baseline evidence was rated as low, with further downgrades due to the bias of selection and methodological heterogeneity. The full summary‐of‐findings (SoF) Table S1 is provided in the Supporting Information.

The CSA of paraspinal muscles is essential for assessing muscle health, particularly in DLS and IS. Many studies have explored the link between CSA, FI, and disease progression, generally agreeing that reduced CSA, especially in deep stabilizers like MF, is associated with atrophy, spinal instability, and worsening slippage [34]. Li et al. [25] found that MF and PM had a significantly higher CSA in healthy controls compared to both IS and DLS patients (*p* < 0.05), reflecting muscle atrophy in patients with spondylolisthesis. Additionally, the ES exhibited a higher CSA in IS patients and controls compared to DLS patients (*p* < 0.05), highlighting more pronounced atrophy in the latter group. FI was also more severe in DLS patients, particularly in the MF and PM, where it was significantly higher than in both IS patients and healthy controls (*p* < 0.05). The ES similarly showed more FI in DLS patients than controls [25].

Cao et al. [29] expanded this by focusing on the role of FI and muscle density in DLS. They found that a decrease in paraspinal muscle density was weakly but significantly correlated with L4 DLS (*p* < 0.05), while the FI ratio was a stronger independent risk factor (OR = 3.746, *p* = 0.038), suggesting that FI, rather than muscle volume or density, might be a better predictor of DLS progression.

Hiyama et al. [21] found a strong correlation between SMM and PM (*p* < 0.001) but also MF with a weaker association (*p* < 0.001). However, no link was found between muscle CSA and the intensity of low back pain. was found suggesting that muscle size might not necessarily be associated with pain levels, further complicating the relationship between muscle degeneration and clinical symptoms.

Lee et al. [23] found that in DLS patients, the MF had a significantly smaller FCSA (244.63 mm^2^ vs. 298.15 mm^2^, *p* = 0.030) and higher FI (56.33% vs. 44.66%, *p* = 0.001) compared to non‐DLS individuals. Conversely, the ES muscle in DLS patients showed a larger FCSA (783.33 mm^2^ vs. 666.22 mm^2^, *p* = 0.028), indicating compensatory hypertrophy. The study concluded that MF atrophy is associated with spinal instability, while ES hypertrophy might serve as a compensatory mechanism to maintain spinal alignment.

Li et al. [17] offered further support for these findings, showing that both DLS and IS patients exhibited significantly higher MF FI and smaller relative FCSA at the L3/L4, L4/L5, and L5/S1 levels compared to healthy controls (*p* < 0.05). The ES muscle, however, showed compensatory hypertrophy in IS patients at the L3/L4 and L4/L5 levels compared to DLS and control groups (*p* < 0.05). This suggests that different muscular adaptations occur in DLS and IS patients, with IS patients showing more hypertrophy in response to instability.

Ohyama et al. [24] found that muscle quality, particularly FI, was better in mild DLS patients compared to non‐DLS individuals. Specifically, the PM and ES had significantly lower FI in DLS patients (*p* = 0.031 for PM and *p* = 0.010 for ES). However, there were no significant differences in the functional or GCSA of the lumbar muscles between the two groups, suggesting that muscle quality might be more important than size in maintaining spinal alignment in mild DLS patients.

Park et al. [22] noted a negative correlation between slippage and both PM and MF mass in IS patients, with greater muscle mass associated with less slippage (*p* = 0.021 for PM, *p* = 0.012 for MF), suggesting that MF plays a protective role in IS, unlike in DLS patients, where muscle mass had less influence on slippage, likely due to age‐related muscle atrophy.

Wagner et al. [20] reported that patients with severe lumbar disability had significantly lower PM CSA compared to those with mild/moderate disability (*p* = 0.041). Larger PM CSA was protective against severe disability (*p* = 0.013), indicating the importance of muscle size in mitigating functional decline [35].

Wang et al. [19] reported that the MF lean CSA (M‐LCSA) was significantly smaller in DLS patients (*p* < 0.01), while the ES lean CSA (ES‐LCSA) was larger, again suggesting compensatory hypertrophy. Additionally, both the MF and ES muscles showed increased T2 signal intensity ratios in the DLS group, indicating higher FI and degeneration. The study concluded that MF atrophy and FI contribute to spinal instability and DLS progression, while ES hypertrophy compensates for this degeneration. According to this concept, Yang et al. [28] found that increased FI was correlated with more severe spondylolisthesis, as measured by the Meyerding grade (*r* = 0.232, *p* < 0.05), emphasizing the role of altered alignment in driving muscle degeneration.

Ding et al. [27] compared DLS and degenerative lumbar kyphosis (DLK) patients and showed that MF atrophy and FI were more pronounced in DLS, especially at lower lumbar levels (L4/L5 and L5/S1), aligning with the segmental instability characteristic of DLS (*p* < 0.05). Interestingly, the PM showed no significant differences in CSA or FI between the two groups (*p* > 0.05). In DLK patients, degeneration was more diffuse in the ES muscle, whereas the MF showed a more significant segment atrophy in DLS patients. These findings may suggest interventions aimed at preserving or enhancing MF function to stabilize the affected segments.

Liu et al. [30] emphasized the role of paraspinal muscle degeneration in the development of single‐segment DLS. The MF showed the most pronounced degeneration, with a significantly reduced relative CSA at L5/S1 (*p* < 0.001), indicating muscle atrophy. FI rate (FIR) of the MF was also significantly higher across all lumbar levels in DLS patients (*p* < 0.001), suggesting extensive fatty degeneration. Similar trends were observed in the ES and PM, with their relative CSA reduced and FIR significantly elevated at all levels (*p* < 0.001).

The study's regression analysis identified FIR in the MF (OR = 1.421, *p* < 0.001) and PM (OR = 1.438, *p* = 0.007) as independent risk factors for DLS, highlighting that FI is a stronger predictor than muscle size reduction. The FIR of the MF showed strong predictive value for DLS (AUC = 0.844). Additionally, a positive correlation between muscle atrophy at L5/S1 and narrowing of the spinal canal at L4/L5 (*p* < 0.05) suggests that muscle degeneration may exacerbate spinal canal narrowing, worsening DLS [30].

Similarly, Wang et al. [26] identified paraspinal muscle atrophy as a key risk factor for DLS. The relative CSA of the MF, ES, and PM was significantly smaller in DLS patients (*p* < 0.05), in line with Liu's findings. Total paraspinal muscle relative CSA was independently predictive of DLS, with a sensitivity of 70% and specificity of 76%, further indicating that muscle atrophy compromises spinal stability and may contribute to vertebral slippage, in opposition to Liu et al. [27] who stated that relative CSA was not a significant predictor for DLS.

In line with these findings, a recent study by Li et al. [31] analyzed that double‐level degenerative LS (dl‐DLS) patients experienced significantly more severe degeneration in their paraspinal muscles compared to single‐level cases (sl‐DLS). In dl‐DLS patients, MF atrophy was more severe from L3–4 to L5–S1, with significantly higher FI across all segments from L1–2 to L5–S1 compared to sl‐DLS patients (*p* < 0.001 for each segment). The pattern observed suggested a segmental progression, with more pronounced atrophy in the lower lumbar segments. The ES exhibited significantly more pronounced atrophy and FI in dl‐DLS patients across all levels from L1–2 to L5–S1 (*p* = 0.035 at L1–2 and *p* < 0.001 for the remaining segments), indicating a diffuse degeneration pattern extending throughout the lumbar spine, without segmental differences between L4–5 and L5–S1 [31].

For the PM, atrophy was significantly greater in dl‐DLS patients from L2–3 to L5–S1 (*p* < 0.001 for each), while FI was significantly higher from L1–2 to L3–4 (*p*‐values ranging from < 0.001 to 0.003) but there were no significant differences in FI at L4–5 and L5–S1. This pattern indicated that PM degeneration was segmental, with more severe changes evident in the upper to mid‐lumbar segments [31].

Across all studies, the majority seem to agree in consistently identifying the MF muscle as the most frequently affected by atrophy [17, 19, 20, 22, 24, 25, 27, 30], considerably impacting spinal stability [19, 20, 21, 22, 24, 31]. The PM [20, 24, 29, 30] and ES [20, 24, 25, 27, 29, 30] also show varying degrees of atrophy, contributing to the overall degeneration of the paraspinal muscle group in both DLS and IS, with different implications for spinal mechanics and progression of slippage.

Alternatively, compensatory hypertrophy of the ES is a common response to spinal instability in both DLS [19, 25] and IS [17, 19]. Li et al. [25] and Lee et al. [23] observed that the ES muscle often enlarges as a way to compensate for the atrophy of the MF, helping to maintain spinal stability. Li et al. [17] similarly noted this hypertrophy in IS patients, where it acts as a stabilizing mechanism, and consistent with the previous observation [25], showing more hypertrophy in the IS group than in the DLS group. Wang et al. [19] further described this compensatory hypertrophy in DLS, suggesting that it plays a role in the body's attempt to mitigate the effects of spinal instability.

Finally FI is a central feature in the degeneration of paraspinal muscles in both DLS [17, 19, 22, 23, 24, 25, 26, 27, 28, 30] and IS, with two studies showing a higher degree of FI in DLS compared to IS [17, 25].

### 4.2 Spinopelvic Parameters and Rehabilitation Strategies

Spinopelvic parameters, including pelvic tilt (PT), pelvic incidence (PI), sagittal vertical axis (SVA), sacral slope (SS), and lumbar lordosis (LL), are essential for understanding spinal biomechanics in degenerative lumbar conditions [28].

Ohyama et al. [24] found that DLS patients exhibited significantly greater upper LL (ULL) compared to non‐DS individuals (*p* = 0.0078).

Yang et al. [28] observed that in DLS patients, those with greater pelvic retroversion (PT > 25°) and larger PI (> 60°) exhibited significantly higher FI in paraspinal muscles (*p* < 0.001), indicating a potential link between altered spinopelvic alignment and muscle degeneration. Similarly, Ding et al. [27] showed that greater FI in the ES, which is essential in maintaining spinal sagittal balance, correlated with reduced LL in patients with DLK (*p* < 0.05). Hiyama et al. [21] found a weak but significant negative correlation between PT and PM CSA at the L4‐L5 level (*p* < 0.05), indicating that decreased muscle mass may influence PT and spinal alignment. This aligns with Li's [31] observations that decreased PM mass influences pelvic alignment. In dl‐DLS patients, PM CSA negatively correlates with PT from L4–5 to L2–3 (*p* < 0.05), indicating that reduced PM mass may be linked to increased PT. Similarly, FI of MF and ES at L5–S1 correlates positively with LL (*p* < 0.05), while in sl‐DLS patients, FI of PM at L4–5 and L5–S1 correlates negatively with LL (*p* < 0.05). For the SVA, a negative correlation exists with ES FI in dl‐DLS from L5–S1 to L1–2 (*p* < 0.05). Li's study [31] expands on this by showing that imbalances between anterior (PM) and posterior (ES) forces may contribute to spinal misalignment, explaining that the PM and ES provide opposing forces with the PM generating anterior shear and ES pulling back. When they degenerate, this imbalance can cause kyphotic or lordotic curvatures, altering PT to compensate for instability. The MF, with reduced stabilizing capacity, further disrupts force distribution, accelerating degeneration and limiting spinal motion [35, 36, 37, 38, 39]. In accordance with these findings, a study representing the state of the art on assessment and conservative treatment emphasizes the importance of prioritizing the restoration of the lumbar MF among local stabilizing muscles [40].

Hence, strengthening and improving the quality of all paraspinal muscles in patients with LS may help stabilize the spine more effectively, potentially reducing the need for surgical intervention and improving outcomes for patients undergoing conservative management.

Disc height plays a crucial role in spinal stability in DLS. In fact, Wang et al. [26] found that the L4/L5 disc height index (DHI) was significantly lower in DLS patients than in healthy controls (*p* < 0.05), indicating advanced degeneration at this level, while the DHI at adjacent levels (L1‐L4 and L5/S1) was significantly higher in DLS patients (*p* < 0.05), suggesting that a greater initial disc height (iDHI) at L4/L5 could be a risk factor for DLS due to increased intervertebral mobility, which promotes vertebral slippage.

Moreover, Wang et al. [26] also revealed a significant reduction in disc height in DLS patients, particularly at anterior and inferior disc levels (*p* < 0.01). Additionally, they noted that lower anterior disc height and MF atrophy were independent protective factors against DLS progression (*p* < 0.05), also highlighting that reduced disc height is a factor in lumbar instability, while a larger initial disc height may increase susceptibility to LS.

### 4.3 Limitations

The findings should be interpreted in the context of the methodological limitations inherent to the primary literature and the review process itself. All included studies used a retrospective, cross‐sectional design, which limits the ability to infer causal relationships between muscle morphology and disease progression.

Furthermore, this review has specific methodological constraints: the restriction to English‐language full‐text articles and limited database searching introduce the potential for language and database bias. The reliance on a narrative synthesis (SWiM) due to heterogeneity requires a cautious interpretation of pooled results. Prospective multicenter studies with larger cohorts and long‐term follow‐up are ultimately needed to confirm the observed associations.

## 5 Conclusion

The review underscores that a decrease in the CSA of deep stabilizing muscles, particularly the MF, is consistently associated with muscle atrophy, spinal instability, and the progression of spondylolisthesis. FI also emerged as a significant factor in disease progression, especially in cases of DLS. Additionally, compensatory hypertrophy of muscles, such as the ES, may occur in response to spinal instability, particularly in IS cases. Understanding these muscle adaptations can guide clinicians in developing personalized rehabilitation strategies aimed at improving patient outcomes and preventing further spinal deterioration. Nevertheless, further well‐designed prospective studies with larger sample sizes are needed to validate these findings and optimize clinical practices.

## Author Contributions

**Carlo A. Mallio:** conceptualization, investigation, funding acquisition, writing – original draft, methodology, validation, visualization, writing – review and editing, software, formal analysis, project administration, resources, supervision, data curation. **Valerio D'Andrea:** conceptualization, investigation, writing – original draft, methodology, visualization, writing – review and editing, software, supervision, data curation, project administration, funding acquisition, validation, formal analysis, resources. **Claudia Volterrani:** investigation. **Paul M. Parizel:** investigation, validation, supervision, formal analysis. **Caterina Bernetti:** methodology, supervision, data curation, visualization. **Federico Greco:** investigation, validation, supervision, visualization. **Bruno Beomonte Zobel:** conceptualization, methodology, formal analysis, supervision. **Johan Van Goethem:** investigation, validation, visualization, formal analysis, supervision. **Gianfranco Di Gennaro:** data curation, project administration, formal analysis, software. **Carlo C. Quattrocchi:** investigation, validation, formal analysis, supervision.

## Funding

The authors have nothing to report.

## Disclosure

The scientific guarantor of this publication is Prof. Carlo Augusto Mallio.

## Ethics Statement

Institutional Review Board approval was not required because this study is a systematic review and does not involve new patient data.

## Consent

The authors have nothing to report.

## Conflicts of Interest

The authors declare no conflicts of interest.

## Supporting information


**Table S1:** Methodological quality and risk of bias assessment using the Newcastle‐Ottawa Scale (NOS). The methodological quality of included observational studies was assessed using the Newcastle‐Ottawa Scale (NOS). The scale evaluates three domains: Selection (Max 4*), Comparability (Max 2*), and Outcome (Max 3*), resulting in a maximum possible score of 9*. Studies are classified as Good Quality (≥ 6*), Fair Quality (4–5*), or Poor Quality (≤ 3*).

## Data Availability

Data sharing not applicable to this article as no datasets were generated or analysed during the current study.
