# Supplementary material for: Systematic Review of Paraspinal Muscle Changes in Lumbar Spondylolisthesis: MRI and CT Insights
Source: Orthop Surg. 2026 Jul 14;18(8):1563–73. doi: 10.1111/os.70362 (PMC13398913; doi:10.1111/os.70362)
Supplement: Supplementary file 1 — Table S1: Methodological quality and risk of bias assessment using the Newcastle‐Ottawa Scale (NOS). The methodological quality of included observational studies was assessed using the Newcastle‐Ottawa Scale (NOS). The scale evaluates three domains: Selection (Max 4*), Comparability (Max 2*), and Outcome (Max 3*), resulting in a maximum possible score of 9*. Studies are classified as Good Quality (≥ 6*), Fair Quality (4–5*), or Poor Quality (≤ 3*). [file OS-18-1563-s001.docx]

**Supplementary Table 1. Methodological Quality and Risk of Bias Assessment using the Newcastle-Ottawa Scale (NOS).** The methodological quality of included observational studies was assessed using the Newcastle-Ottawa Scale (NOS). The scale evaluates three domains: Selection (Max 4*), Comparability (Max 2*), and Outcome (Max 3*), resulting in a maximum possible score of 9*. Studies are classified as Good Quality (≥ 6*), Fair Quality (4-5*), or Poor Quality (≤3*).

| Study (Author, Year) | Selection  (Max 4*) | Comparability  (Max 2*) | Outcome  (Max 3*) | Total Score | Overall Quality |
| --- | --- | --- | --- | --- | --- |
| Li et al. (2024a) [17] | ⋆⋆⋆⋆ | ⋆⋆ | ⋆ | 7/9 | Good |
| Ohyama et al. (2021) [27] | ⋆⋆⋆ | ⋆⋆  (Age/Sex Matched) | ⋆ | 6/9 | Good |
| Li et al. (2022) [20] | ⋆⋆⋆ | ⋆  (Age Match, Adjusted) | ⋆ | 5/9 | Fair |
| Lee et al. (2021) [25] | ⋆⋆ | ⋆ (  Adjusted Factors) | ⋆ | 4/9 | Fair |
| Park et al. (2019) [29] | ⋆⋆ | ⋆  (Internal Comparison) | ⋆ | 4/9 | Fair |
| Hiyama et al. (2019) [32] | ⋆⋆ | ⋆  (Adjustment for Age/Sex) | ⋆ | 4/9 | Fair |
| Cao et al. (2023) [28] | ⋆⋆ | ⋆  (Adjustment for Age/Sex) | ⋆ | 4/9 | Fair |
| Wang et al. (2015) [19] | ⋆⋆ | ⋆ | ⋆ | 4/9 | Fair |
| Wang et al. (2022) [30] | ⋆⋆ | ⋆ | ⋆ | 4/9 | Fair |
| Ding et al. (2022) [21] | ⋆⋆ | ⋆ | ⋆ | 4/9 | Fair |
| Yang et al. (2023) [26] | ⋆⋆ | ⋆ | ⋆ | 4/9 | Fair |
| Liu et al. (2024) [22] | ⋆⋆ | ⋆ | ⋆ | 4/9 | Fair |
| Li et al. (2024b) [33] | ⋆⋆ | ⋆ | ⋆ | 4/9 | Fair |
| Wagner et al. (2018) [31] | ⋆⋆ | ⋆ | ⋆ | 4/9 | Fair |
| Li et al. (2024a) [17] | ⋆⋆ | ⋆ | ⋆ | 4/9 | Fair |

**Summary-of-Findings (SoF) Table: Certainty of Evidence for Key Morphological Associations using the GRADE System**

This table presents the main associations found in the systematic review and their corresponding certainty of evidence, as evaluated by the **GRADE** (Grading of Recommendations Assessment, Development and Evaluation) methodology. Evidence certainty is rated as High, Moderate, Low, or Very Low.

| **Key Association/Outcome** | **Number of Studies (N=14)** | **Effect/Magnitude of Association** | **Certainty of Evidence (GRADE)** | **Rationale for Rating & Downgrades** |
| --- | --- | --- | --- | --- |
| **MF Degeneration**  **(Association between SL and MF atrophy/FI)** | 14  (Table 1) | **Consistent Finding:** MF shows decreased CSA and significantly increased FI in SL groups vs. controls. FI ratios often 10%–30% higher than controls. | Low | **Initial Rating:**  Low (Observational/Retrospective designs).  **Downgrades:**  Risk of Bias (moderate-to-high risk across studies, lack of full blinding/matching).  Inconsistency/Imprecision (wide range of reported effect sizes and methodological heterogeneity in FI measurement). |
| **Compensatory ES Hypertrophy**  **(Association between IS and ES CSA maintenance/increase)** | 6  (e.g. Li et al. (2024a) [17]) | **Observed Finding:** ES CSA/rCSA is often maintained or increased in IS patients compared to DLS or controls. | Low | **Initial Rating:**  Low (Observational designs).  **Downgrades:**  Risk of Bias (lack of adjustment for all confounders).  Inconsistency (some DLS studies show ES atrophy, others show compensation).  Indirectness (hypertrophy is often compromised by high FI, thus compensation is functionally ineffective). |
| **Functional Quality as Predictor**  **(Prognostic value: association between FI and SL progression/risk)** | 5  (e.g., Lee et al. (2021) [25], Cao et al. (2023) [28]) | **Prognostic Finding:** High FI (especially MF FI Ratio) is an independent risk factor (e.g., OR = 3.746 for high PM FIR in L4 DLS). Lower MF FCSA is an independent protective factor for DLS. | Very Low | **Initial Rating:** Low (Observational designs).  **Downgrades:**  Risk of Bias (high selection bias, confounding by age/BMI).  Imprecision (wide confidence intervals for ORs).  High Suspected Publication Bias (only studies showing statistically significant risk factors may be published). |
